# Validation and Psychometric Properties of the Chinese Version of the Fear of Missing Out Scale

**DOI:** 10.3390/ijerph18189896

**Published:** 2021-09-20

**Authors:** Yan-Yu Li, Yi-Ting Huang, Kai Dou

**Affiliations:** Department of Psychology and Research Center of Adolescent Psychology and Behavior, School of Education, Guangzhou University, Guangzhou 510006, China; 2111808175@e.gzhu.edu.cn (Y.-Y.L.); 1666100038@e.gzhu.edu.cn (Y.-T.H.)

**Keywords:** fear of missing out, measurement invariance, psychometric properties, validation, mobile phone addiction, life satisfaction

## Abstract

Objectives: The present study aimed to examine the psychometric properties of Przybylski’s 10-item Fear of Missing Out scale and to investigate the measurement invariance across age and gender among Chinese adolescents and emerging adults. Methods: A convenient sample of 2886 subjects (M_age_ = 14.79, SD = 4.03; 47.96% male) was recruited in south China. Item analysis, EFA, CFA, testing of measurement invariance across gender and age, reliability analysis, and concurrent validity analysis were conducted. A subsample of 154 subjects (M_age_ = 15.3, SD = 2.22; 54% male) completed the scale again after 6 months to assess the test–retest reliability. Results: The EFA strongly indicated a two-dimensional solution, including fear of missing novel information and fear of missing social opportunities, for the Chinese version of the FoMO scale, which the CFA confirmed. Adequate internal consistency was found. Concurrent validity and discriminant validity were also demonstrated. Conclusions: Based on the results, the Chinese version of the FoMO scale is suitable for use among young Chinese people.

## 1. Introduction

Fear of missing out (FoMO) is a concept describing a general feeling of fear that others might have a more satisfying experience than the subject when they are absent, and it is characterized by a strong desire to stay with others [1]. FoMO has received great attention from researchers due to the fact of its frequent manifestation and unique contribution in associating with a higher engagement of social media use [2,3]. For example, a recent study, using a representative Polish sample, found that all respondents experienced some level of FoMO, yet to varying degrees [4]. Specifically, 81% of the respondents reported experiencing moderate to high levels of FoMO. In addition, one recent meta-analysis of FoMO underscored that the correlation between FoMO and social media use was stronger than the correlations between other variables (e.g., loneliness, depression, and stress) and social media use [5]. According to the latest national report [6], approximately 0.99 billion people in China used the Internet, and 99.2% of them used social media in their daily lives. While social media has brought great convenience to individuals’ lives, it may also impact their physical and mental health when used problematically [7]. Therefore, alleviating FoMO is a possible and practical way to lower the risk of problematic social media use, and it is necessary to explore and assess FoMO in China.

In the literature, since FoMO was firstly studied academically in 2013 [1], several scales have been developed to measure it. Przybylski et al. (2013) [1] firstly developed a 10-item and single-factor scale measuring individuals’ experience of general FoMO. Subsequently, Alt (2015) [8], based on Przybylski’s FoMO scale, developed a 10-item FoMO scale consisting of three dimensions (i.e., social media engagement, news information engagement, and commercial information engagement). More recently, Zhang et al. (2020) [9] developed an 8-item scale consisting of two dimensions (i.e., personal and social FoMO). To measure some specific kinds of FoMO, Wegmann et al. (2017) [10] developed a 12-item and two-dimensional scale, assessing trait-FoMO and state-FoMO. Additionally, Abri (2017) [11] developed a scale measuring FoMO in an alcohol-related context. In China, some Chinese researchers have also developed FoMO scales in a Chinese context. Song et al. (2017) [12] developed a scale measuring FoMO in the context of mobile social media use. Lately, Ma et al. (2021) [13] considered FoMO as negative emotion and developed a 15-item scale consisting of four dimensions (i.e., missing motivation, missing cognition, missing emotion, and missing behavior). Among these scales, the lack of validity analysis causes the low likelihood of most scales being used more widely. The most popular and widely used scale is the scale developed by Przybylski in 2013 [1]. Therefore, Przybylski’s FoMO scale may be a more suitable assessment tool to measure individuals’ general FoMO in China.

Przybylski’s FoMO scale, the most widely used validated instrument in psychological studies to measure individuals’ general FoMO [3,14], is a self-report questionnaire developed by Przybylski and his colleagues [1] based on self-determination theory [15]. It has been translated into Turkish [16,17], Arabic [18], Spanish [19], and Italian [20]. The results of previous studies showed that the reliability of internal consistency and test–retest reliability of different versions were good but that the structural validity was inconsistent. Specifically, the Turkish [16,17] and the Spanish [19] versions showed a one-factor structure supporting the original English version structure with good internal consistency. However, the Italian [20] version showed a two-factor structure, and the data fit a two-dimensional model better with proper internal consistency. Similarly, the Arabic [18] version had a two-factor solution with good internal consistency, but only 8 items met the psychometric requirements.

Findings regarding the measurement invariance across genders were inconsistent. On the one hand, Elhai et al. (2018) [21] tested measurement invariance of Przybylski’s FoMO scale across genders among college students and found higher factor loadings for women respondents, indicating that the FoMO construct may have slightly different meaning and interpretation between genders. However, the findings should be interpreted with caution due to the discrepant sample sizes between genders. On the other hand, Casale et al. (2020) [20] tested gender invariance and found that the measurement model is comparable in both genders.

Regarding the concurrent validity of Przybylski’s FoMO scale, previous studies have found that FoMO is not only positively associated with online problematic behaviors, such as social media disorder [22], smartphone addiction [23], and problematic internet use [14], but also has associated with psychopathological symptoms [24] and leads to negative consequences such as more phubbing behavior [2], poorer academic performance [25], more sleep problems [26], and lower well-being [27]. Therefore, it was expected that FoMO is positively associated with smartphone addiction but negatively associated with subjective well-being.

The present study aimed to examine the psychometric properties of Przybylski’s FoMO scale in a Chinese context. This study is of great importance for the reason that it affords further cross-cultural research on the FoMO scale and a practical instrument to measure FoMO in China. However, it is necessary to note that Przybylski’s FoMO scale has already been translated and used in China. For example, Xie et al. (2018) [28] firstly translated it and studied the predicting role of basic psychological need satisfaction on FoMO and its underlying mechanism among Chinese undergraduates. Afterward, a handful of researchers used the translated scale among different sample groups in China such as high school students [5], college students [2], and middle school students [29]. Unfortunately, all these studies used this early translated version without knowing its psychometric characteristics, only reporting their Cronbach’s α, and data on dimensionality, reliability, and validity were still unknown. Therefore, we translated the scale and examined its reliability and validity. Moreover, the present study also investigated measurement invariance across gender because previous studies obtained inconsistent findings regarding measurement invariance across genders [20,21]. In addition, the present study also investigated the measurement invariance across age (i.e., adolescents and university students), repeating the previous research and extending it by examining participants aged from 8 to 27 [20].

## 2. Methods

### 2.1. Participants and Procedures

A convenience sample of 3064 participants from primary schools, middle schools, and universities in South China was recruited between March and December 2019. However, 178 participants did not complete the survey and were excluded. The final sample comprised 2886 respondents whose ages ranged from 8 to 27 years (*M_age_* = 14.79, *SD* = 4.03; 47.96% male; 18.6% university students). The sample was randomly split into halves using the algorithm available in IBM SPSS Statistics 21, and the two halves were named sample 1 (*n* = 1443; *M_age_* = 14.77, *SD* = 4.03; 49.10% male) and sample 2 (*n* = 1443; *M_age_* = 14.79, *SD* = 4.05; 46.50% male). There were no significant differences in gender, age, or the total score (*ts* = 0.01~1.39) between the two samples (*ps* > 0.05). Moreover, a subsample of 154 participants (*M_age_* = 15.3, *SD* = 2.22; 54% male), named sample 3, completed the Chinese version of the FoMO scale again after a 6-month time interval to examine the test–retest reliability.

Prior to data collection, written informed consent was obtained from all individual participants included in this study. A notice was given to them that included a description of the study’s purposes and assurance that personal data would remain confidential and anonymous. Concerning the participation of children and adolescents, we also sought written consent from their legal guardians. Participants completed the surveys in the paper-and-pencil format during their classes in the presence of a research assistant. The study procedures were approved by the Ethics Review Committee (IRB) of Guangzhou University (No. GZHU2019007).

### 2.2. Measure

#### 2.2.1. Fear of Missing Out Scale-Chinese Version (FoMOs-C)

Przybylski’s FoMO scale is a self-reported questionnaire with 10 items (e.g., I fear others have more rewarding experiences than me), with a higher score indicating a higher level of FoMO [1]. Items were rated on a 5-point Likert scale from 1 (not at all true for me) to 5 (extremely true for me). The original English version of the FoMO scale was translated into Chinese using the back-translation method. Two bilingual researchers translated the initial draft, and then another two experienced individuals translated the version back to English. Minor discrepancies were settled through consensus. The reliability and validity of the scale are described in later sections of this paper.

#### 2.2.2. Mobile Phone Addiction Index (MPAI)

Smartphone addiction was measured using a Chinese version of the MPAI [30,31]. 17 items (e.g., You always feel that there is not enough time to use your smartphone) were rated on a 5-point Likert scale from 1 (never) to 5 (always), with higher scores indicating higher addiction severity. In this study, Cronbach’s α was 0.90, indicating adequate reliability.

#### 2.2.3. Satisfaction with Life Scale (SWLS)

The SWLS was used in this study to measure the degree of overall life satisfaction [32]. Five items (e.g., I am satisfied with my life) were rated on a 7-point Likert scale from 1 (strongly disagree) to 7 (strongly agree). The Chinese version of the SWLS has good validity and internal reliability [33]. In this study, Cronbach’s α was 0.87, indicating adequate reliability.

### 2.3. Data Analysis

Statistical analysis consisted of several steps including (1) item analysis, (2) exploratory factor analysis (EFA), (3) confirmatory factor analysis (CFA), (4) measurement invariance testing, and (5) assessment of concurrent validity and reliability. All data analyses were performed using IBM SPSS Statistics 21. Measurement invariance testing and CFA were conducted with Mplus 8.

First, item analysis and EFA were conducted with sample 1. For item analysis, the item-total correlation test and critical ratio method were used. For EFA, we conducted a principal component analysis with Varimax rotation. Second, CFA was conducted with sample 2. For CFA, the model fit to the data was assessed by the Chi-square statistic, comparative fit index (CFI > 0.90), Tucker–Lewis index (TLI > 0.90), standardized root means square residual (SRMR < 0.08), and root mean square error of approximation (RMSEA < 0.06) [34]. In addition, composite reliability, average variance extracted was conducted to demonstrate the reliability of the measure. The composite reliability was acceptable when larger than 0.60 [35]. The average variance extracted was a relatively conservative estimate of the validity of the measure [36]. Third, measurement invariance testing was performed on sample 1 and sample 2 to examine whether FoMO scores were invariant across gender and age at the configural, factor loadings, and intercept levels. Measurement invariance was examined by carrying out multigroup CFA. The change in CFI was considered to indicate a good fit when below 0.01 (ΔCFI < 0.01) [37,38]. Finally, Cronbach’s alpha, test–retest reliability, and concurrent validity were analyzed. A Cronbach’s α value ≥ 0.70 was considered acceptable [39].

## 3. Results

### 3.1. Item Analysis

First, the item-total correlation test was performed, and the results showed that all 10 items had good psychometric properties (*r* = 0.51~0.71, *p* < 0.001). Moreover, the critical ratio method was used to analyze the degree of discrimination of the items in the scale. All the respondents were ranked according to their total scores from high to low. Then, those whose scores were in the top 27% were classified as the high score group (*n* = 779), and those whose scores were in the bottom 27% were classified as the low score group (*n* = 779). The independent sample *t*-test was used to compare the differences between the two groups of subjects for each item. The results showed that all 10 items had good psychometric properties (*CR* = 20.71~50.10, *p* < 0.001). Therefore, we retained all 10 items to conduct EFA.

### 3.2. Construct Validity

#### 3.2.1. Exploratory Factor Analysis (EFA)

The KMO value was 0.82 for the 10-item FoMOs-C. Bartlett’s test of sphericity showed that the correlation matrix was suitable for factor analysis (*χ^2^* = 5457.89, *df* = 45, *p* < 0.001). The EFA of the FoMOs-C produced two significant factors that had eigenvalues greater than 1, and that explained a total of 56.03% of the variance. Item 5 (It is important that I understand my friends “in jokes”) was deleted due to the low factor loadings on both factors (below 0.4). The remaining 9 item factor loadings were between 0.42~0.90 and explained a total of 59.87% of the variance. Item 4 (I get anxious when I don’t know what my friends are up to) was deleted due to the high factor loadings on both factors (above 0.4). The remaining 8 item factor loadings were between 0.49~0.91 and explained a total variance of 61.48%. Finally, as shown in Table 1, the first factor consisted of four items, while the second factor contained another four items; the eigenvalues were 3.46 and 1.46, and the proportions of explained variance were 43.29% and 18.20%, respectively. The scree plot provides a graphic representation of these results and indicates a two-factor solution of the FoMOs-C. There was a moderate linear correlation between the two factors (*r* = 0.44, *p* < 0.01). The two factors were termed “fear of missing novel information” (FoM-NI) and “fear of missing social opportunities” (FoM-SO). The result was different from that of the original single-factor English version.

#### 3.2.2. Confirmatory Factor Analysis (CFA)

**Figure 1:**
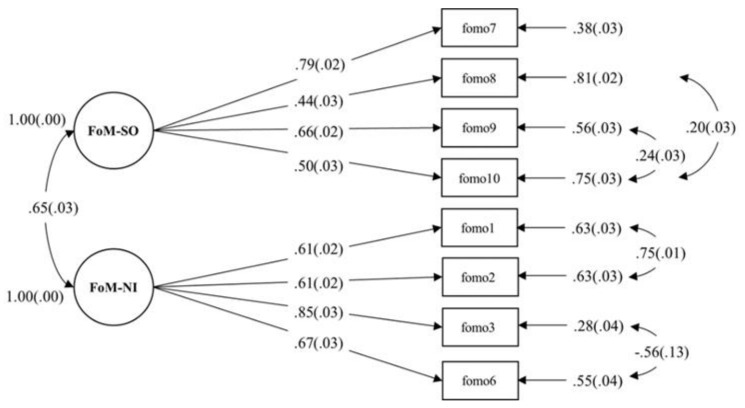
Path diagram for the CFA of the Fear of Missing Out Scale-Chinese version. FoM-SO represents fear of missing social opportunities; FoM-NI represents fear of missing novel information.

To verify the factor structure identified through EFA, CFA was conducted. Considering the Chinese culture, we, following the modification indices suggestions, added the error covariance between item 1 and item 2, item 3 and item 6 (referring to factor 1), item 8 and item 10, and item 9 and item 10 (referring to factor 2). After we added the error covariance, the results showed that all fit indexes met statistical standards, indicating that the structure of the FoMOs-C with two factors and 8 items had a good fit and appropriate structural validity. Considering the inconsistent results regarding the factor structure, we carried out comparisons among the two-factor structure in the current study (8 items without item 5 and item 4), the one-factor structure in the original study (10 items), and the two-factor structure in the Arabic study (8 items without item 5 and item 8). The results showed that the structure of the FoMOs-C with two factors and 8 items in the current study fit the data better than the other two structures (see Table 2 and Figure 1). In addition, the results indicated that the average variance extracted for factor 1 (FoM-SO) was 0.38, the composite reliability was 0.70; the average variance extracted for factor 2 (FoM-SI) was 0.48, the composite reliability was 0.78, which met the acceptable level suggested by Fornell and Larcker (1981) [35] and Lam (2012) [36].

#### 3.2.3. Concurrent Validity and Discriminant Validity

We assessed concurrent validity and discriminant validity by examining the bivariate correlations between the two FoMOs-C factor scores and other measures. As reported in Table 3, the results showed that the FoMOs-C had good concurrent validity and discriminant validity.

### 3.3. Measurement Invariance

Tests of configural invariance and metric invariance were sequentially conducted across ages with 2349 adolescents and 537 university students for FoMOs-C. As shown in Table 4, the results showed that configural and metric invariances were established across adolescents and university students. Specifically, the result indicated an adequate fit to the data of the configural model, thus demonstrating that the same pattern of common fixed and free parameters was held across groups. The results of the metric model showed that the ΔCFI value between the configural model and metric model was not greater than 0.01, which indicated factor loading invariance across age. When the intercepts were constrained to be equal, the ΔCFI value between the metric model was greater than 0.01.

In addition, measurement invariance across genders was assessed with 1375 males and 1492 females for the FoMOs-C. As shown in Table 4, the result demonstrated that the configural and metric invariances were established across genders. Specifically, the result of the configural model indicated an adequate fit overall. The ΔCFI value between the configural model and metric model was not greater than 0.01, which indicated factor loading invariance across gender.

### 3.4. Reliability

To test the reliability of the FoMOs-C, the two factors were separately subjected to internal consistency tests with the total sample. The Cronbach’s α for the whole scale was 0.82, and those for factors 1 and 2 were 0.80 and 0.74, respectively. The intraclass correlation coefficient (ICC) was used to estimate the test–retest reliability. The test–retest reliability coefficient of the whole FoMOs-C was 0.63, and those for factors 1 and 2 were 0.55 and 0.66 (*p* < 0.001), respectively. The results suggested the satisfactory reliability of the FoMOs-C.

## 4. Discussion

The present study examined the factorial structure and reliability of the FoMOs-C to psychometrically test a tool that assesses general FoMO among Chinese young people. The factor analysis identified a two-factor solution that accounted for 61.48% of the total variance. The CFA confirmed that the two-factor solution had an adequate fit, which is consistent with Li’s study [40] and Arabic [18] and Italian [20] versions of the FoMO scale. In the current study, the two factors were named “fear of missing novel information” (FoM-NI) and “fear of missing social opportunities” (FoM-SO) based on the item content and Chinese cultural context. FoM-NI includes items related to a general apprehension that others may have more rewarding experiences than the subject or have fun without the subject. It reflects people’s need for the latest information because they like to “spend time keeping up with what is going on”. Moreover, FoM-NI explains most of the total variance, which suggests that it has excellent convergence with the original conceptualization of FoMO. Moreover, the final four items in FoM-SO reflect the individual’s need for interaction and connection.

In EFA, we removed item 4 and item 5. First, the removal of item 5 was consistent with the Arabic version of the FoMO scale [18] and Li’s study [40]. Li et al. (2019) [40] suggested that there are two possible reasons for this consistency. On the one hand, the subjects of Arabic, Li’s, and the current study were young people, while the study in which the original version of the FoMO scale was developed included adults. It is possible that adults may fear being excluded from the group because they do not understand “in jokes” making them feel disconnected from society. Therefore, item 5 may be more adapted to adults, and future studies should examine this possibility. On the other hand, both Arabia and China are in Asia, and their cultural backgrounds are mainly collectivist. The concept of FoMO was initially born in the Western context and considering the cultural differences between the East and the West, item 5 may be more applicable in the Western cultural context. Moreover, the loadings of item 4 on the two factors were above 0.4. For the conciseness of the model, we decided to remove this item. In addition, item 4 is the only item on the scale that refers to anxiety, and FoMO is a concept that refers more to a general apprehension than a pathology [20], which suggests that item 4 is not suitable for the scale. In CFA, we tested the model fit of the scales. The results indicated that compared with the one-factor scale and Arabic version of the scale, the FoMOs-C showed a better model fit, verifying good construct validation.

The measurement invariance across gender and age, which may be considered a prerequisite for making quantitative comparisons, provides essential support for the validity of the FoMOs-C. In this study, both gender invariance and age invariance were supported at the level of configural and metric invariance. The results indicate that the measurement model was comparable across genders and age groups, consistent with previous findings [20]. Findings regarding measurement invariance suggested that the construct validity of the FoMOs-C was high.

The FoMOs-C also showed good concurrent validity. Significant and positive correlations were found between FoMOs-C scores and smartphone addiction, consistent with previous studies [23,41]. Significant and negative correlations were found between FoMOs-C scores and subjective well-being, supporting previous findings [1,27].

The 8-item FoMOs-C is a useful and effective instrument with good psychometric properties for assessing FoMO among Chinese adolescents and university students. This is the very first study to revise the most widely used FoMO scale into a Chinese version for a youth sample. In practice, there are two important ways to apply the scale. Regarding the sample in the current study, most previous research focused on an adult population [1,20]. Nevertheless, the revised Chinese scale is more suitable for adolescents and the emerging adult population under the Chinese cultural context. Additionally, there were two dimensions of the revised Chinese version scale, which were named “fear of missing novel information” (FoM-NI) and “fear of missing social opportunities” (FoM-SO). Compared with children and adults, adolescents have a higher level of sensation-seeking and risk-taking due to the unbalanced development in the brain [42]. They are more likely to be afraid of not keeping up with their peers and, therefore, fear missing the updated information. Meanwhile, relationships with family and peers have always been crucial and associated with adolescent’s psychological functioning [43]. Adolescents would develop attachment security and social capital from social opportunities [43]. Therefore, the practicality of the revised Chinese scale for adolescence study could lead to further understandings of the underlying psychological mechanisms for FoMO.

However, several limitations must be considered in the interpretation of these results. First, the use of the convenience sampling technique may result in all data being collected in a small area in south China, which prevents the results from being generalized to the general Chinese population. Moreover, because the study focused on students, generalizing results to nonstudents may not be warranted. Future studies should be carried out on more representative samples to justify the current findings. Second, for better database management, participants were asked to write down their names when doing the survey, and all data were collected using self-report questionnaires. Because we handled all data anonymously only at the data analysis stage, it may still be subject to social desirability bias even though participants were informed that personal data would remain confidential and anonymous. Therefore, future studies should use more objective methods, for example, collecting data using the experience sampling method or examining FoMO using experimental design to provide more robust insights into the level of FoMO. Third, further studies should be conducted to strengthen the validity of the scale. For instance, future studies should evaluate the relationships between FoMO and other variables highly associated with FoMO, for example, different forms of social media use, because a substantial amount of research reveals that FoMO is the most vital risk factor of social media use and that individuals with a high level of FoMO are prone to use smartphones for a social purpose [5,29,44].

## 5. Conclusions

Despite the limitations, the present findings indicated that the FoMOs-C is a useful instrument with good psychometric properties for assessing FoMO among young people in China. This study is critical because it enriches cross-cultural research on FoMO scales and provides a validated tool to measure FoMO in China. More specifically, the FoMOs-C has high construct validity, good reliability, and demonstrable measurement invariance.

## Figures and Tables

**Table 1:** Factor analysis of the Fear of Missing Out scale-Chinese version.

Factors	1	2
Factor 1		
I fear others have more rewarding experiences than me.	0.91	
I fear my friends have more rewarding experiences than me.	0.91	
I get worried when I find out my friends are having fun without me.	0.72	
Sometimes, I wonder if I spend too much time keeping up with what is going on.	0.49	
Factor 2		
It bothers me when I miss an opportunity to meet up with friends.		0.67
When I have a good time, it is important for me to share the details online (e.g., updating status).		0.65
When I miss out on a planned get-together it bothers me.		0.78
When I go on vacation, I continue to keep tabs on what my friends are doing.		0.79
Eigenvalue	3.46	1.46
% of the variance explained	43.29	18.20

KMO = 0.79, Bartlett’s Test = 4288.51, *df* = 28, *p* < 0.001.

**Table 2:** Comparison of fit indices for three FoMO scales factor solutions.

Model	*χ* *^2^*	*df*	RMSEA	CFI	TLI	SRMR
1 Factor	1849.24	35	0.19	0.67	0.57	0.10
2 Factors (Arabic version) ^a^	722.87	19	0.16	0.85	0.78	0.10
2 Factors (Chinese version)	68.07	15	0.05	0.99	0.98	0.02

^a^ Al-Menayes et al. [18].

**Table 3:** The means, standard deviations, and correlations among the scales.

	*M*	*SD*	1	2	3	4
1. FoMOs-C	2.59	0.73	-			
2. FoM-NI	2.50	0.84	0.83 ***	-		
3. FoM-SO	2.68	0.90	0.86 ***	0.43 ***	-	
4. MPAI	2.12	0.67	0.39 ***	0.36 ***	0.30 ^***^	-
5. SWLS	3.92	1.30	−0.13 ***	−0.21 ***	−0.01	−0.24 ***

*** *p* < 0.001.

**Table 4:** Measurement invariance of FoMOs-C across age and gender.

Model	*χ* *^2^*	*df*	RMSEA	CFI	TLI	ΔCFI
Age						
Model 1 configural invariance	157.96	32	0.05	0.98	0.97	-
Model 2 metric invariance	175.82	38	0.05	0.98	0.97	<0.01
Gender						
Model 1 configural invariance	118.66	28	0.05	0.99	0.98	-
Model 2 metric invariance	131.65	34	0.05	0.99	0.98	<0.01

## Data Availability

The data presented in this study are available on request from the corresponding author.
